# Low fatness, reduced fat intake and adequate plasmatic concentrations of LDL-cholesterol are associated with high bone mineral density in women: a cross-sectional study with control group

**DOI:** 10.1186/1476-511X-11-37

**Published:** 2012-03-12

**Authors:** Karin S Sarkis, Lígia A Martini, Vera L Szejnfeld, Marcelo M Pinheiro

**Affiliations:** 1 Nutrition Department, School of Public Health, Sao Paulo University, Av. Dr. Arnaldo, 715, São Paulo-SP, Brazil CEP-01246-904; 2 Nutrition Department, School of Public Health, Sao Paulo University, Av. Dr. Arnaldo, 715, São Paulo - SP, Brazil CEP-01246-904; 3 Rheumatology Division, Universidade Federal de São Paulo/Escola Paulista de Medicina (Unifesp/EPM), Av Dr Altino Arantes, 669, apto 105, Vila Clementino, São Paulo-SP, Brazil CEP 04042-033; 4 Rheumatology Division, Universidade Federal de São Paulo/Escola Paulista de Medicina (Unifesp/EPM), Rua Pedro de Toledo, 650-2° Andar-Vila Clementino, CEP 04039-002 São Paulo-SP, Brazil, Disciplina de Reumatologia

**Keywords:** Bone mineral density, Body composition, Lipid metabolism, Diet, Mineral metabolism, Women

## Abstract

**Background:**

Several parameters are associated with high bone mineral density (BMD), such as overweight, black background, intense physical activity (PA), greater calcium intake and some medications. The objectives are to evaluate the prevalence and the main aspects associated with high BMD in healthy women.

**Methods:**

After reviewing the database of approximately 21,500 BMD scans performed in the metropolitan area of São Paulo, Brazil, from June 2005 to October 2010, high BMD (over 1400 g/cm^2 ^at lumbar spine and/or above 1200 g/cm^2 ^at femoral neck) was found in 421 exams. Exclusion criteria were age below 30 or above 60 years, black ethnicity, pregnant or obese women, disease and/or medications known to interfere with bone metabolism. A total of 40 women with high BMD were included and matched with 40 healthy women with normal BMD, paired to weight, age, skin color and menopausal status. Medical history, food intake and PA were assessed through validated questionnaires. Body composition was evaluated through a GE-Lunar DPX MD + bone densitometer. Radiography of the thoracic and lumbar spine was carried out to exclude degenerative alterations or fractures. Biochemical parameters included both lipid and hormonal profiles, along with mineral and bone metabolism. Statistical analysis included parametric and nonparametric tests and linear regression models. *P *< 0.05 was considered significant.

**Results:**

The mean age was 50.9 (8.3) years. There was no significant difference between groups in relation to PA, smoking, intake of calcium and vitamin D, as well as laboratory tests, except serum C-telopeptide of type I collagen (s-CTX), which was lower in the high BMD group (*p *= 0.04). In the final model of multivariate regression, a lower fat intake and body fatness as well a better profile of LDL-cholesterol predicted almost 35% of high BMD in women. (adjusted R2 = 0.347; *p *< 0.001). In addition, greater amounts of lean mass and higher IGF-1 serum concentrations played a protective role, regardless age and weight.

**Conclusion:**

Our results demonstrate the potential deleterious effect of lipid metabolism-related components, including fat intake and body fatness and worse lipid profile, on bone mass and metabolism in healthy women.

## Background

In recent years, the occurrence of high bone mineral density (BMD) has been observed in some individuals [1]. However, little is known about the prevalence, physiopathogenic aspects involved, associated risk factors or the clinical relevance of this entity in medical practice, including the possibility to be a variant of normality or be considered a disease itself, since it associates with some morbidities.

So far, there is controversy about the definition of individuals with high BMD, since the World Health Organization [2] criteria for classification of osteopenia and osteoporosis does not consider this condition. Thus, individuals with values above one standard deviation (SD) are considered as normal, regardless of the absolute value or the number of standard deviations above the unit.

Although there are few studies on this subject, most of them point out to the role of anthropometric data (overweight and obesity) [3] and demographics (males and black ethnicity) [4], as well as genetic factors (LRP5 mutations) [5], lifestyle habits (intense physical activity [6], higher calcium intake above 1500 mg/day and food or water with excess fluoride) [7,8], as the main factors positively associated with high BMD. Some medications such as statins [9] and thiazide diuretics [10] may also have a protective effect. In addition, patients with breast and endometrial cancer [11], type II diabetes mellitus [12] and athletes have higher BMD than healthy individuals [13].

Thus, a better understanding of these aspects can help to optimize the management of patients with osteoporosis as well as minimize the burden of osteoporotic fractures throughout the world.

The objective of this study was to identify the prevalence, as well as risk factors and protection associated with high BMD in healthy women, through cross-sectional study with control group.

## Results

Clinical and nutritional data (mean ± SD) are listed in Table 1. The groups were matched for age, weight, BMI, smoking, fat mass and menopausal status. In both groups, the prevalence of overweight was high, but without significant difference between them. Nevertheless, women with high BMD had higher FFM and ALM (*p *< 0.05). Seven healthy control women (17.5%) and three (7.5%) of the high BMD group were classified as sarcopenic (*p *= 0.31).

**Table 1:** Anthropometric, clinical and nutritional data of the population, according to BMD

	HBMD (N = 40)	Healthy controls (N = 40)	p
Age (years)	50.9 (8.2)	51 (8.5)	0.989

Weight (kg)	64.3 (5.2)	61.7 (6.7)	0.052

Height (m)	1.58 (0.1)	1.57 (0.0)	0.241

BMI (kg/m**^2^**)	25.6 (2.4)	25.2 (2.6)	0.497

Age of menarche (years)	13.0 (2.4)	13.0 (1.4)	0.913

Parity	2.4 (1.4)	2.2 (1.3)	0.460

Smoking packets-year	16.7 (19.3)	26.2 (27.7)	0.290

TPA index (score)	7.6 (1.2)	7.9 (1.3)	0.412

Bone Mineral Density			

Lumbar spine (g/cm**^2^**)	1.408 (0.1)	1.209 (0.1)	< 0.001

T-score	1.9 (1.0)	0.2 (0.8)	< 0.001

Z-score	2.5 (1.1)	0.9 (0.9)	< 0.001

Femoral neck (g/cm**^2^**)	1.119 (0.1)	0.986 (0.08)	< 0.001

T-score	0.9 (0.9)	-0.2 (0.6)	< 0.001

Z-score	1.7 (0.6)	0.7 (1.0)	< 0.001

Total femoral (g/cm**^2^**)	1.166 (0.1)	1.043 (0.2)	< 0.001

T-score	1.4 (1.0)	0.2 (0.7)	< 0.001

Z-score	1.9 (1.1)	0.7 (0.7)	< 0.001

Body Composition			

FFM (kg)	36.4 (4.1)	34.4 (3.9)	0.029

ALM (kg)	16.1 (1.9)	15.0 (1.7)	0.007

RSMI (kg/m**^2^**)	6.3 (0.7)	6.1 (0.7)	0.278

Body fat (%)	39.4 (6.8)	40.5 (6.0)	0.433

Total body fat mass (kg)	24.0 (4.8)	23.6 (5.4)	0.740

Dietary Intake (day)			

Energy (kcal)	1697.3 (460.5)	1749.3 (575.9)	0.653

Protein (g)	74.2 (10.9)	67.0 (11.3)	0.005

Fat (g)	57.6 (11.9)	58.8 (14.9)	0.700

Carbohidrate (g)	235.1 (30.7)	238.7 (36.9)	0.637

Calcium (mg)	836.4 (249.8)	754.7 (219.7)	0.124

Phosphorus (mg)	1178.9 (178.8)	1068.2 (153.9)	0.004

Vitamin D (μg)	1.9 (2.2)	2.0 (1.4)	0.944

*BMI *Body Mass Index, *TFA *Total Physical Activity, *FFM *Total Fat-Free Mass, *ALM *Appendicular Lean Mass, *RSMI *Relative Skeletal Muscle Index, Nutrients adjusted to energy. T- student Test

In the high BMD group, the only significant correlations found were between femoral neck BMD and BF% (r = -0.36; *p *= 0.02), BF (r = -0.45; *p *= 0.004) and RSMI (r = 0.35; *p *= 0.02).

After the adjustment of food intake for energy, significant difference was obtained between the groups regarding the intake of protein and phosphorus, with greater levels in women with high BMD (*p *< 0.05). The mean daily consumption of lipids, carbohydrates and vitamin D was similar in both groups. The mean intake of macronutrients as well as phosphorus were within the DRI values proposed by the Food and Nutrition Board [14-16]. Conversely, the mean calcium and vitamin D intake were below the proposed recommendations (Table 1).

While no statistically significant difference of metabolic, hormonal and bone parameters was observed between the groups, the serum concentration of total cholesterol was above the recommended values and more vitamin D insufficiency was found in the control group (Table 2). In this group, only four women (10%) reported 25-OH-D concentrations lower than 20 ng/mL.

**Table 2:** Metabolic, hormonal and biochemical parameters of the metabolism bone and mineral of the population study, according BMD

	HBMD (N = 40)	Healthy controls (N = 40)	p
Glucose (mg/mL)	94.2 (20.0)	88.4 (17.4)	0.17
Uric acid (mg/dL)	4.8 (1.4)	4.5 (1.1)	0.40
Cholesterol total (mg/dL)	192.0 (45.3)	205.5 (38.6)	0.16
LDL-Cholesterol (mg/dL)	114.0 (32.4)	124.0 (33.2)	0.18
HDL-Cholesterol (mg/dL)	58.4 (13.2)	60.9 (9.5)	0.33
VLDL-Cholesterol (mg/dL)	22.3 (11.3)	21.8 (10.3)	0.86
Triglyceride (mg/dL)	111.9 (57.6)	109.9 (51.3)	0.82
Leptin (ng/mL)	0.4 (0.2)	0.4 (0.2)	0.78
IGF-1 (ng/mL)	115.7 (53.1)	134.5 (56.6)	0.13
Total testosterone (ng/dL)	22.8 (9.9)	26.9 (16.6)	0.59
Prolactin (ng/mL)	13.3 (15.6)	8.0 (4.6)	0.14
FSH (mUI/mL)	55.7 (50.9)	72.6 (51.5)	0.15
LH (mUI/mL)	18.2 (2.9)	20.2 (3.2)	0.09
Serum alkaline phosphatase (U/L)	108.9 (57.7)	113.3 (74.2)	0.77
Serum total calcium (mg/dL)	9.2 (0.4)	9.3 (0.4)	0.06
Serum ionic calcium (mmol/L)	1.2 (0.1)	1.2 (0.1)	0.66
Serum magnesium (mg/dL)	2.0 (0.2)	2.0 (0.2)	0.84
Serum phosphorus (mg/dL)	3.5 (0.5)	3.6 (0.5)	0.49
Serum CTX (ng/mL)	0.16 (0.2)	0.24 (0.3)*	0.04
Serum Vitamin D (ng/mL)	33.0 (15.7)	29.9 (9.5)	0.28
Serum PTH intact (pg/mL)	31.5 (14.9)	31.6 (15.4)	0.97
Urinary sodium (mEq/24h)	140.8 (68.2)	151.0 (58.9)	0.50
Urinary phosphorus (mg/24h)	381.7 (347.1)	432.4 (368.2)	0.56
Urinary calcium (mg/24h)	110.1 (84.7)	109.4 (103.4)	0.98

*IGF-1 *insulin-like growth factor, *FSH *follicle-stimulating hormone, *LH *luteinizing hormone, *CTX *carboxy-terminal collagen crosslinks; *Leptin, Prolactin, CTX and Vitamin D were transformed in log for performed the statistical analysis*; Student's t-test

In patients with high BMD, negative correlation was verified between femoral neck BMD and total cholesterol (r = -0.30; *p *= 0.01) and LDL-cholesterol (r = -0.39; *p *= 0.01). On the other hand, serum IGF-1 correlated positively with lumbar spine BMD (r = 0.36; *p *= 0.02). Similar correlation was also observed between 25-OH-D and RSMI (r = 0.33; *p *= 0.04) and leptin and BF (r = 0.44; *p *= 0.004). Bone resorption was lower in high BMD women (*p *< 0.05) than in controls (Table 2).

In the final multiple linear regression model, IGF-1 was positively correlated with lumbar spine BMD in women with high BMD after adjustments for age, weight, BMI, menopause, smoking, lean mass and fat and total energy of the diet. In contrast, LDL-cholesterol and body fatness associated negatively while RSMI and femoral neck BMD associated positively. Additionally, higher fat intake as well as greater serum phosphorus and iPTH had negative association with total body BMD (Table 3).

**Table 3:** Final model of multivariable regression using equations to estimate HBMD in women

Equations	R	R^2^	p
Spine BMD = 1.305+(0.361)IGF-1	0.361	0.108	0.022
Femur BMD = 1.096-0.272(TBFM)-0.396(LDLC)+0.337(RSMI)	0.659	0.347	< 0.001
Total body BMD = 1.599-0.330(FI)-0.413(P)-(0.298)PTH	0.649	0.367	< 0.001

*IGF-1 *insulin-like growth factor, *TBFM *total body fat mass, *LDLC*, LDL- cholesterol, *RSMI*, relative skeletal muscle index, *BMD*, body mineral density, *FI *fat intake, *P *serum phosphorus, *iPTH *intact serum parathyroid hormone; Models adjusted for age, weight, height, BMI, smoking, lean mass, fat mass, energy intake and menopause

## Discussion

Our results show that the prevalence of women with high BMD in the healthy general population is relatively low (2%) and that the main positive aspects are independently associated with IGF-1 and skeletal lean mass. Conversely, aspects related to fats, such as higher body fatness, increased plasmatic concentration of LDL-cholesterol and fat intake, are associated negatively with the BMD in these individuals.

To our knowledge, this is the first study that has identified the main risk factors and protection associated with high BMD in healthy women from the general population, including the three components of body composition and nutritional aspects, as well as biochemical, mineral and hormonal parameters.

It is important to note that there is still not a clear definition of high BMD by the scientific community. In the most recent (2007) official positions of the International Society for Clinical Densitometry (ISCD) [17], there are no cut-off points (T- or Z-score) to classify the patient with high BMD [18]. Some authors use Z-score above 2.5 DP [19] and others consider the absolute value of BMD (g/cm^2^) in its largest interquartile range [1]. In this study, a T-score above + 2 SD, in the absence of fractures or other osteosclerosing disorders, was chosen for this classification. These values were defined in accordance with the premise that T-score values below - 2 SD are indicative of low BMD and increased risk of fractures [17]. Thus, T-score values above + 2 SD could be used to classify individuals with high BMD.

IGF-1 works in both bone formation and resorption [20] and thus plays a relevant role for the acquisition and maintenance of bone mass. Looking at our data of healthy adult women, there was significant association with high BMD, especially in trabecular bone, explaining nearly 11% of the bone density variation in these individuals. Other authors have found similar results [21-23].

Although there is vast positive evidence of IGF on bone mass, its function should be better explored. Recently, Cohen et al. showed osteoblasts seem to be resistant to the IGF-1 effect in women with osteoporosis. Thus, it would not have a positive role and should be evaluated with caution [24]. Furthermore, Clemens & Karsenty, underscore osteocalcin as a regulator of glucose metabolism by means of insulin receptors present in osteoblasts, acting both on systemic glucose homeostasis and BMD increment [25].

Lean mass is the main component of body composition associated with BMD [26-29], in which mechanical stress [30] and mechanosensory signaling, promoted by osteocytes, are the most studied determinants [31]. These aspects have been partially confirmed by our results since physical activity, per se, was unable to explain high BMD in healthy women.

Most likely, the women with high BMD evaluated in our study had lower bone resorption rate, as suggested by the lower values of CTX when compared to healthy controls. In view of the negative correlation between this bone turnover marker and the RSMI (r = -0.31; *p *= 0.05), we suggest that the trophic role of muscle mass (pro formation), associated with lower bone resorption, resulted in a positive net effect on BMD. One of the possibilities to explain lower bone resorption in women with high BMD may be lower serum FSH- and LH-plasma concentrations (*p *= 0.09), which inherently can play a negative role on bone tissue [32,33].

Alternatively, there are conflicting studies on the role of fat mass on BMD [34,35], since body weight traditionally has a positive role on bone mass, especially with greater cortical and periosteal remodeling [35]. In our case, fat mass, including percentage and absolute values, played a negative role on femoral neck BMD, independent of body weight, hormonal variables (aromatization of estrone to estradiol), and adiposity type (gynecoid vs. android).

The physiopathogenic mechanisms involved with the deleterious role of body fatness on bone tissue are not fully known, but some authors believe that protective bone strength is most associated with dynamic loads, those found with lean and muscular mass, than with static, observed with fat mass [36]. Moreover, the proinflammatory state observed in individuals with obesity or with greater adiposity may be related to increased bone resorption and thus greater injury to bone health, introducing the new concept of lipotoxicity [37].

It is important to emphasize that, according to our data, there was positive correlation between BF and serum leptin concentrations, suggesting greater peripheral resistance to leptin and therefore to insulin. Recently, leptin has been pointed out as an important regulator of osteoclast differentiation, since it controls the expression of RANKL (receptor activator of nuclear factor kappa-B ligand) and CART (cocaine and amphetamine regulated transcript) [38].

Worse lipid profile, defined by LDL cholesterol, was another aspect negatively associated with femoral neck BMD in women with high BMD, emphasizing once again the participation of lipid metabolism on bone tissue [35]. Lipoprotein lipid oxidation is able to stimulate osteoclastogenesis [39], as well as the greater intake of fats, via RANKL expression on activated T lymphocytes [40]. Moreover, this oxidation may have a negative action on osteoblast differentiation and bone formation [41]. Recently, some authors have shown greater bone loss [42] and increase in resorption bone markers in patients with hypercholesterolemia [43].

Although the incidence of dyslipidemia in patients with osteoporosis is still not known, the atherogenic lipid profile is known to be significantly associated with lower bone density in postmenopausal women [44]. However, the use of statins does not seem to play a beneficial role on bone health [45].

Likewise, the daily intake of lipids was also a factor negatively associated with BMD, as previously reported by other authors [46]. The ingestion of large amounts of fat can negatively affect the absorption of calcium, prostaglandin synthesis and osteoclastogenesis. It also increases the oxidation of lipids [47]. In addition, the ingestion of large amounts of fat is related to the increased expression of adipocyte differentiation factors, in particular to PPAR-γ (peroxisome proliferator-activated receptor gamma) [48]. According to data from the Framingham Osteoporosis Study, high fat consumption can be harmful to bone mass, particularly in individuals with greater allelic variation in PPAR-γ [48]. In our study, no gene polymorphisms were evaluated. It is worth mentioning that the exclusion of obese women reinforces our findings, since, in all likelihood, they consumed even more fat. Thus, lipid consumption above DRI recommendations is able to negatively affect BMD.

Traditionally, higher concentrations of iPTH and phosphorus are associated with worse bone health, as observed in patients with chronic renal insufficiency. Our results show that this combination is associated negatively with BMD in patients with high BMD.

Surprisingly, no significant difference was observed in mean plasma concentrations of vitamin D between the groups. Accordingly, women had high BMD independent of vitamin D, while on the other hand there was positive interaction between vitamin D, lean mass and higher femoral neck BMD. The role of vitamin D receptor (VDR) in muscle cells [49] could explain these findings. Unfortunately, falls, muscle strength and physical incapacity were not objectives of this study and further research is needed to better understand the interaction between these aspects.

Additionally, women with high BMD had higher intake of protein and phosphorus, after adjustments for energy. However, when adjusted for protein intake, there was no significant difference in the daily intake of phosphorus (1144 .1 ± 140.3 vs. 1103.6 ± 123.8 mg, *p *= 0.17) between the high BMD and control groups, respectively. Several epidemiological observations on protein intake confirm our results [50], although some have shown a negative role [51]. When observing the beneficial role of protein intake, it is important to note that the effect of the protein-induced acid load does not promote bone loss or urinary calcium [52]. In addition, protein intake is able to increase IGF-1 values by almost 30% [53] and thus provide beneficial effect on bone metabolism. The combination of these two latter aspects was observed in our study.

Conversely, when evaluating protein intake (g) adjusted for body weight (g/kg), there was no significant difference between groups (1.2 g/kg for high BMD vs. 1.1 g/kg in controls, *p *= 0.2), although both were above the recommendation for healthy individuals (0.8 g/kg).

The limitations of this study include the lack of evaluation of genetic markers, the lack of measurement of muscle strength and variables that quantify the state of oxidative stress, as well as the amount of fluoride in water or in food ingested and the type and the quantification of visceral fat.

The strengths of this study included the careful selection of the sampling procedures, excluding the risk factors traditionally associated with high BMD such as black ethnicity, obese individuals, athletes and patients infected by hepatitis C virus (HCV) or with degenerative alterations of the lumbar spine or hip. Moreover, results were strengthened with the inclusion of a control group matched for age, weight, fat mass, ethnicity, physical activity and smoking, which reduces various biases and confounding factors.

This study expands upon the scientific understanding of bone and mineral metabolism, since it includes new aspects of practical interest, such as body adiposity, lipid profile and fat intake, and the non-pharmacological handling of patients with bone fragility. Thus, beyond adapting the intake of calcium and vitamin D and stimulating resistance exercises for an individual with osteoporosis, the physician and nutritionist should also guide the lower intake of fats, encourage aerobic activities and improve the lipid profile of these individuals.

## Conclusion

Thus, our results show that the main protection factors associated with high BMD in healthy women are IGF-1 plasmatic concentration, skeletal lean mass and intake of protein. On the other hand, body fatness, worse lipid profile and fat intake played a negative role.

## Methods

### Study design, sampling and patient selection

After reviewing the database of approximately 21,500 BMD scans performed in healthy women for any reason, from June 2005 to October 2010, were found 421 exams (1.96%) with high BMD. This information has been originated from the São Paulo metropolitan area, Brazil, including primary, secondary and tertiary hospitals as well as data from general practitioners.

Through convenience sampling and consecutively, exams with BMD values above 1400 g/cm^2 ^or T-score greater than + 2 SD at lumbar spine and/or above 1200 g/cm^2 ^or T-score greater than + 2 SD at femoral neck were eligible to this study. Furthermore, none of these sites could have osteopenia or osteoporosis [2] or previous fracture.

The control group included healthy women matched for age, weight and ethnicity. Besides, they had BMD values lower than 1400 g/cm^2 ^(T-score less than 1.99 DP) at lumbar spine and/or under 1200 g/cm^2 ^(T-score less than 1.99 DP) at femoral neck. Similarly, none of the sites could have osteopenia or osteoporosis [2].

Women aged below 30 and above 65 years, black people, pregnant and those with BMI higher than 29.9 kg/m^2 ^were excluded. Moreover, the presence of diseases associated with any nutritional imbalance or osteometabolic conditions, including renal, infectious, neoplastic, digestive, endocrinologic, rheumatic and cardiovascular diseases were not included. History of alcoholic consumption [54], dyslipidemia, chronic hepatitis C, hormone treatment and individuals using statins or thiazide diuretics also were not eligible for the study.

Of 421 women, 381 (90.5%) were excluded for not meeting the eligibility criteria, particularly obesity and black ethnicity. Thus, 40 (9.5%) women constituted the study group.

The study protocol was examined and approved by the Research Ethics Committee of the University of São Paulo (USP) (number: 0170/09) and Universidade Federal de São Paulo (Unifesp/EPM) (number: 0229/04) and the participants who agreed to participate in the study gave written informed consent.

### Collection of clinical information

Standardized questionnaires were used to verify the demographic characteristics and medical history. Menopause was defined as more than 12 months since the last menstrual period [55].

### Evaluation of food intake

Food intake was measured through a Food Frequency Questionnaire (FFQ), consisting of 62 food items and validated in Brazil [56]. The FFQ was administered by a trained nutritionist and the information provided reflected the pattern of food intake for the last six months. Portion size was evaluated with the aid of a food portion photo set.

Dietsys software, version 4.0 (National Cancer Institute, Bethesda, MD), was used to calculate the daily nutritional intake of total energy (kcal/day), macronutrients and some micronutrients, including calcium, phosphorus and vitamin D. All nutrients were adjusted for energy. Phosphorus was also adjusted to protein intake according to the method proposed by Willett & Stampfer [57] and compared with the Dietary Reference Intakes (DRIs) recommendation from Food and Nutrition Board [14-16].

### Evaluation of physical activity

Physical activity (PA) was assessed through the Baecke Questionnaire of Habitual Physical Activity [58]. This tool evaluates three PA's main components: work, sport and leisure. All the answers, with the exception of the occupation activity and the type of sport, were precoded on a scale of 5 points, with descriptors that range from never (1) to very often (5). The level of occupational activity was classified as low (1), medium (3) or high (5). The score of sport activities was calculated by the equation: [intensity code × duration code × year proportion code] × 1.25. If there was more than one sport practiced, the values were added. As a result, each component of PA can receive a maximum of five points, with a maximum score of 15. Each index was rounded to the nearest tenth of the unit or point.

### Anthropometry

All individuals were measured and weighed on a standard balance beam scale (Filizola^®^), calibrated periodically, wearing light clothes and without shoes. Weight was measured to the nearest 100 g. Standing height was measured with the aid of a stadiometer (Filizola^®^) by a trained individual. Body mass index (BMI) was calculated by the ratio between weight (kg) and height, in meters squared (kg/m^2^).

### Assessment of body composition and bone mineral density measurements

BMD assessment was performed on the lumbar spine (L1-L4) and femoral neck (g/cm^2^) by using dual energy X-ray absorptiometry (DXA), DPX MD + densitometer (GE-Lunar Radiation Corporation, Madison, WI, USA). A well-trained technician followed a standard protocol. Quality control was done daily and phantom cross-calibration three times per week. For premenopausal women, Z-score was used in compliance with International Society of Clinical Densitometry (ISCD) recommendations [21] and supported by the Brazilian Society of Clinical Densitometry (SBDens), a national regulatory agency [59].

Body composition was also measured by DXA. Total body fat mass was evaluated in absolute values as body fat (kg) and percentage (BF%). Lean mass was defined as fat free mass (FFM), with special emphasis to appendicular lean mass (ALM) and the relative skeletal muscle index (RSMI). For women aged over 50 years, sarcopenia was defined according to the classification of Baumgartner [60], in which RSMI values below two standard deviations relative to young healthy population or less than 5.45 kg/m^2^, for women; indicative of that condition. For younger women, the criterion was not used. RSMI is the ratio of appendicular lean mass (kg) and height squared (m^2^).

The coefficient of variation was 1.5% for the lumbar spine and total body and 2% for the femoral neck.

### Radiographic evaluation

Radiographic evaluation of dorsal and lumbar spine in anteroposterior and lateral positions was performed by a rheumatologist blinded for clinical data and specific procedures of the protocol data in order to exclude vertebral degenerative processes, as well as other causes of high BMD, including bone metastases, Paget's disease, fracture or congenital deformities (Schmorl's nodes, severe scoliosis). The image acquisition protocol followed the recommendation of 120 cm for tube film distance and X-ray beams centered in T8 and L3, respectively.

### Laboratory and biochemical analysis

All the blood samples were collected in the morning after twelve hours of fasting.

Metabolic and hormonal parameters included glucose and uric acid, both evaluated by calorimetry; total cholesterol, fractions and triglycerides by the Trinder method; insulin growth factor-1 (IGF-1) by chemiluminescence, leptin by ELISA (IBL International) and total testosterone by RIA. Prolactin, follicle-stimulating hormone (FSH) and luteinizing hormone (LH) were measured by fluorometric method.

Bone and mineral metabolism were also measured. Bone formation was evaluated by alkaline phosphatase (ALP) activity through enzymatic-kinetic method. Bone resorption was assessed by serum C-terminal fragment of type-I collagen (CTX), through chemiluminescence. Serum total calcium, ionized calcium and magnesium were measured by colorimetric assay and serum phosphorus by UV mobility. Intact parathyroid hormone (iPTH) and 25-hydroxivitamin D (25-OH-D) were evaluated by electrochemiluminescence. Additionally, 24-hour urine was collected for measuring urinary calcium by colorimetric assay, as well as the fraction of sodium excretion (potentiometry) and phosphorus (UV mobility).

The classification of vitamin D sufficiency adopted was proposed by Dawson-Hughes [61].

### Statistical analysis

The results were analyzed as mean ± SD. The Kolmogorov-Smirnov test was used to verify the normal distribution of variables. Data with non-normal distribution were transformed into logarithm. Mean differences between groups were assessed by Student's t-test. Categorical variables were analyzed by chi-square test of association. The correlation between variables was evaluated by parametric tests and Pearson correlation.

Multiple linear regression models were used to identify factors associated with high BMD, used as dependent variable. Clinical characteristics, food intake data, physical activity score, body composition measurements and concentrations of biochemical parameters were considered as independent. P-values of < 0.05 were considered significant. All analyses were performed with the aid of Statistical Package for the Social Sciences, version 16.5 for Windows (SPSS Inc., Chicago, IL).

## Competing interests

No disclosures. There is no affiliation, financial agreement or any other involvement with any company.

## Authors' contributions

**KSS **performed the sample collection, nutritional assessments and processed the data, as well as conducted statistical analysis and drafted the manuscript. **VLS **and **LAM **participated in the design of the study and helped in analyzing data and in drafting the manuscript. **MMP **participated in the design of the study, performed medical appointment and BMD measurements and analyzed all spine X-ray exams. Additionally, he helped in data interpretation and in drafting of the manuscript. All authors have read and approved the final version.
